# Experimental superposition of orders of quantum gates

**DOI:** 10.1038/ncomms8913

**Published:** 2015-08-07

**Authors:** Lorenzo M. Procopio, Amir Moqanaki, Mateus Araújo, Fabio Costa, Irati Alonso Calafell, Emma G. Dowd, Deny R. Hamel, Lee A. Rozema, Časlav Brukner, Philip Walther

**Affiliations:** 1 Faculty of Physics, University of Vienna, Boltzmanngasse 5, Vienna A-1090, Austria; 2 Institute for Quantum Optics and Quantum Information, Austrian Academy of Sciences, Boltzmanngasse 3, Vienna A-1090, Austria

## Abstract

Quantum computers achieve a speed-up by placing quantum bits (qubits) in superpositions of different states. However, it has recently been appreciated that quantum mechanics also allows one to ‘superimpose different operations'. Furthermore, it has been shown that using a qubit to coherently control the gate order allows one to accomplish a task—determining if two gates commute or anti-commute—with fewer gate uses than any known quantum algorithm. Here we experimentally demonstrate this advantage, in a photonic context, using a second qubit to control the order in which two gates are applied to a first qubit. We create the required superposition of gate orders by using additional degrees of freedom of the photons encoding our qubits. The new resource we exploit can be interpreted as a superposition of causal orders, and could allow quantum algorithms to be implemented with an efficiency unlikely to be achieved on a fixed-gate-order quantum computer.

## Abstract (web summary)

It has been conjectured that not only states but also quantum operations can be placed in a superposition of causal order. Here, the authors use a qubit superpose the order in which two photonic gates are applied, which is shown to enable a more efficient detection of their commutation relations.

## Graphical abstract

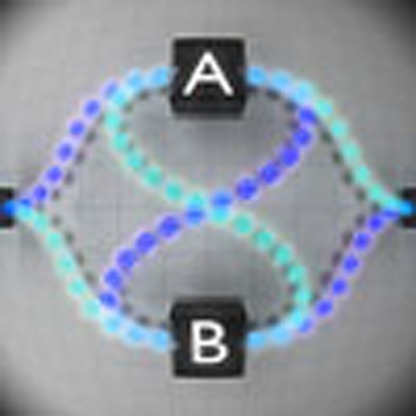

Quantum mechanics has long been recognized as a counter-intuitive theory, with ideas such as wave-particle duality, quantum superposition and entanglement defying our natural way of thinking. In recent years, these sorts of uniquely quantum properties are being exploited to develop revolutionary technologies, such as quantum cryptography, quantum metrology and perhaps the most well-known example, quantum computation. In the field of quantum computation, the circuit model was used to show that universal quantum computation is possible^1^, and the circuit model has since been an incredibly successful tool, leading to important quantum algorithms which greatly outperform their classical counterparts^2^. The circuit model takes advantage of the fact that quantum mechanics allows for the superposition and interference of quantum bits (qubits) in different states to achieve a computational speed-up. However, in addition to the superpositions of states, quantum mechanics also allows for the superposition of quantum circuits^3^,^4^—a feature which is not used in the standard quantum circuit model. Nevertheless, such superpositions of quantum circuits are rapidly becoming central to several foundational research programs studying the role of time and causality in quantum theory^5^,^6^,^7^,^8^,^9^. These superpositions of quantum circuits (sometimes called a ‘superposition of causal orders') give rise to new counter-intuitive quantum predictions, and it has recently been predicted that they could provide quantum computers with even further computational advantages^8^,^10^. In particular, superimposing quantum circuits, each with a different gate ordering, can allow one to accomplish a specific computational task with fewer quantum gate uses than a quantum computer which has a fixed-gate order^10^.

One of the most useful methods for quantifying the performance of a quantum algorithm is its query complexity. Loosely speaking, this is the number of times that a quantum gate is used (or queried). The use of the query complexity is motivated by the assumption that applying a gate is a cost that we wish to minimize. In an optical quantum computer this cost would be either another physical copy of the gate (say a different set of waveplates, or interferometer), or a repeated usage of the same gate at a later time. In an ion-trap^11^ or super-conducting^12^ quantum computer the cost would be the application of another pulse sequence to the qubits. Given that one of the main difficulties in creating a scalable quantum computer is the implementation of multiple gates^13^,^14^, techniques to reduce the query complexity are essential for practical quantum computing.

Just as in a classical electronic circuit, in a fixed-order quantum circuit one connects a series of logic gates by wires (see Fig. 1a or Fig. 1b). This is an intuitive and extremely powerful method for designing quantum algorithms, but there are advantages to other models. One example is measurement-based quantum computing^15^,^16^, a different paradigm than the circuit model which paved the way for many experimental implementations of quantum algorithms^17^,^18^. Unlike other models of computation, what is considered here is a strict extension of the quantum circuit model, which therefore allows for additional computational power. The particular extension we study in our experiment is to allow for superpositions of different quantum circuits; that is, to coherently control which quantum circuit is applied on an input state (see Fig. 1c). In this case, the order of quantum gates acting on a set of qubits could be controlled by the state of another set of qubits—this is not allowed in the standard-quantum circuit model, wherein the gate order is independent of the state of the qubits^4^.

Coherently controlling the order of quantum gates conditioned on the state of a set of qubits is a new type of operation. A proposal for one such operation is the ‘N-SWITCH', which takes *N* different gates and applies them in a given superposition of different permutations^19^. Using this operation, a quantum algorithm has recently been proposed to solve a specific problem with a query complexity of *O*(*N*), while a fixed-order circuit is likely to require *O*(*N*^2^) queries to solve the same problem^9^,^10^. In other words, the N-SWITCH reduces the query complexity by a factor *N*. Since it has been shown that the N-SWITCH can be simulated by *N*^2^ fixed-order gates^10^, a factor of *N* is the maximum advantage that can be achieved with this operation. It is an open question whether there exist other resources without fixed-gate order that would allow further advantages.

In this paper, we report on our experimental demonstration of a 2-SWITCH operation, which we implement by taking advantage of additional degrees of freedom of the physical system which encodes our qubits. Our technique makes it possible to apply two quantum gates in a superposition of both possible orders, and it enables us to determine if the gates commute or anti-commute with only a single use (or query) of each gate. On the other hand, determining if two gates commute or anti-commute using a quantum circuit with a fixed-gate order would require at least two uses of one of the gates^8^,^20^. Moreover, when this problem is scaled to *N* gates, creating a superposition of quantum circuits is likely to provide an exponential advantage over classical algorithms, and a linear advantage over quantum algorithms with fixed-gate order^10^. The techniques that we demonstrate here could allow some quantum algorithms to be implemented with an efficiency that is unlikely to be achieved on a quantum computer with a fixed-gate order.

## Results

### Theoretical objective

**Figure 1:**
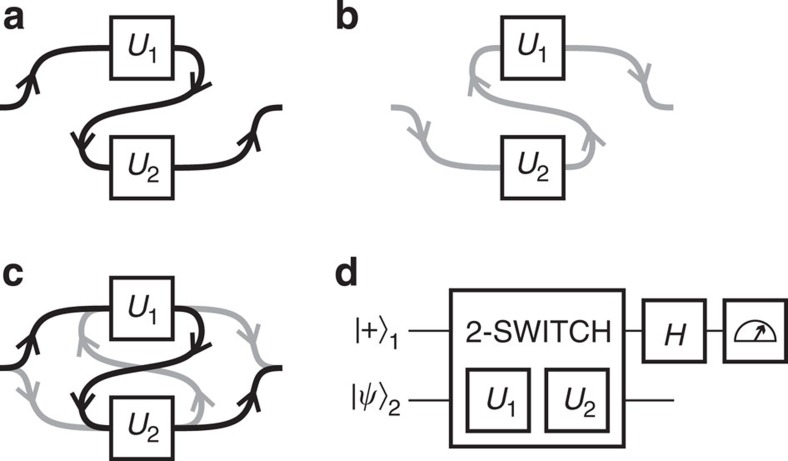
Theoretical Concept. (**a**) Given two unitary gates, *U*_1_ and *U*_2_, the circuit model allows us to wire them in one of two possible ways: either *U*_1_ before *U*_2_, or (**b**) *U*_2_ before *U*_1_. (**c**) Quantum mechanics allows us to coherently control both options, such that the qubit sees both *U*_1_ before *U*_2_, *and U*_2_ before *U*_1_. (**d**) The 2-SWITCH operation applies *U*_1_ and *U*_2_ to qubit 2 in both orders, as shown in panel c, dependent on the state of qubit 1. Unless at least one of *U*_1_ and *U*_2_ is used more than once, the 2-SWITCH operation *cannot* be implemented with standard circuit-model elements. To be explicit, the 2-SWITCH applies *U*_1_*U*_2_ to 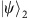 (the lower qubit) if the upper qubit is in 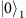, and *U*_2_*U*_1_ to 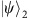 if the upper qubit is in 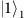. Measuring the state of qubit 1 in the 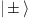 basis allows one to unambiguously decide if *U*_1_ and *U*_2_ commute or anti-commute with only a single use of each gate. In this circuit, H is the Hadamard gate, and 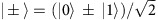.

In our task, one is presented with two unitary gates, *U*_1_ and *U*_2_, and the guarantee that *U*_1_ and *U*_2_ either commute or anti-commute (but *U*_1_ and *U*_2_ are otherwise unknown and arbitrary). The goal is to determine which statement is true. This is the first step towards demonstrating the quantum algorithm proposed in ref. 10, and, in the case with *N*=2 causal orders, it corresponds to the information processing task introduced in ref. 8. In the standard-circuit model this task cannot be carried out with a single use of each gate^8^; this limitation is evident in all previous experimental investigations of commutation relations, which all used one gate at least twice^21^,^22^. However, using the 2-SWITCH operation to apply *U*_1_ and *U*_2_ in a superposition of both orders allows us to distinguish whether the gates commute or anti-commute with only one use of each gate. To see this, consider using the 2-SWITCH operation in the circuit shown in Fig. 1d. Given that the 2-SWITCH applies *U*_1_*U*_2_ if the upper qubit is in 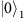 and *U*_2_*U*_1_ if upper qubit is in 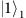, it is straightforward to show that, when the input to the circuit is initially in the state 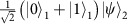 (where 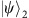 is an arbitrary state of qubit 2), the result (after performing a Hadamard gate on the control qubit, and before measuring it) is the state


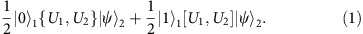


In equation (1), [*U*_1_, *U*_2_] and {*U*_1_, *U*_2_} are the commutator and anti-commutator of *U*_1_ and *U*_2_, respectively. Given the guarantee of either commutation or anti-commutation, if the upper qubit is measured and found in 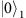 we know for certain that the gates commute; on the other hand, if it is found in 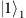 we know for certain that the gates anti-commute. Thus one can unambiguously distinguish between the two cases. Note that, although Fig. 1d shows the 2-SWITCH operation as a gate in a quantum circuit, it cannot be implemented by querying *U*_1_ and *U*_2_ only once in a fixed order.

### Experimental implementation

Although creating superpositions of circuits is a conceptually simple idea, it is not immediately clear how it could be carried out in the laboratory. The most obvious solution is to place the physical circuit elements—such as wires or optical fibres connecting the gates—in a quantum superposition. However, this would require quantum control over macroscopic systems, and is likely to remain unattainable in the foreseeable future. Instead, we use additional degrees of freedom of our qubits to control the order with which they traverse the gates. Note that other implementations have been independently proposed, both making use of internal degrees of freedom (G. Chiribella, R. Ionicioiu, T. Jennewein and D. Terno, personal communication, July 2012)^10^,^23^,^24^, and in adiabatic quantum computing^25^. The internal degrees of freedom could be any degrees of freedom of the physical system that the qubits are encoded in. For example, trapped ions possess many electronic and vibrational modes, many of which could be suitably controlled^23^. In our experiment, we use a spatial degree of freedom of photonic qubits to create a superposition of different gate orders acting on a qubit encoded in the photon's polarization. While the use of multiple degrees of freedom of a single photon is a well-known tool in photonic-quantum computing^26^,^27^,^28^,^29^,^30^,^31^,^32^, our work is the first experimental demonstration which uses this tool to superimpose quantum gate orders. Furthermore, using controlled-path gates^31^, which coherently place a photon in one of two paths dependent on the state of another photon, our technique would form the basis of a 2-SWITCH operation between two different photons, making it possible to create multi-particle circuits with no fixed-gate order.

**Figure 2:**
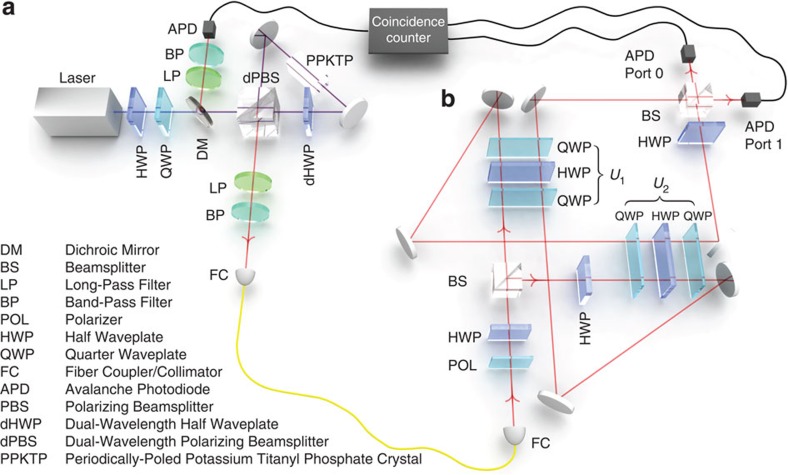
Experimental Implementation. Our optical implementation to distinguish whether a pair of unitary gates commute or anti-commute with only a single copy of each gate. The photons for our experiment are generated in a separable polarization state using a Sagnac source (**a**). One photon is used as a herald, and the second is fed into the interferometer (**b**). The unitary gates in question are each implemented with three waveplates, and act on the polarization of single photons.

At the centre of our implementation is a Mach–Zehnder interferometer with a loop in each arm (see Fig. 2), which allows us to create the required superposition of gate orders. In particular, it enables one qubit (qubit 1 of the circuit in Fig. 1d), encoded in a spatial degree of freedom of the photon, to coherently control the order in which two gates are applied to another qubit (qubit 2 of Fig. 1d), encoded in the photon's polarization. We choose to use a spatial degree of freedom as control (that is different than previous theoretical work which proposed using polarization as control^10^,^23^) since it proved to be much more stable experimentally. Briefly, a single photon is sent to a 50/50 beamsplitter, which creates the spatial qubit: 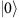 if transmitted, and 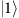 if reflected. The unitary gates *U*_1_ and *U*_2_ are implemented on the polarization state of the same photon using a set of waveplates. Now, dependent on whether the photon is reflected or transmitted, the polarization qubit will see either *U*_1_*U*_2_ or *U*_2_*U*_1_. The two paths then coherently recombine on a final 50/50 beamsplitter (enacting the Hadamard gate shown in Fig. 1c). Finally, simply measuring the state of the spatial qubit (that is, whether the photon exits port 0 or 1 of the final beamsplitter) tells us if *U*_1_ and *U*_2_ commute or anti-commute.

Before continuing, we must specify what constitutes a ‘gate use' in a photonic experiment. In accordance with previous work^10^,^23^, we define a single gate use as a single photon passing through a single physical copy of a gate. This definition is well motivated, since it quantifies both the number of physical devices required, and the experimental cost of rerouting photons through the same gate at a later time. If we imagine affixing counters to each set of waveplates, which increment every time a photon passes by, then we can read the number of uses of a gate directly off of the counter. Note that such a counter system will factorize out, and thus will not destroy the interference^10^. In this hypothetical situation, each time a photon passes through our experiment the counters of *U*_1_ and *U*_2_ will always read 1. Thus, by this definition, we use each gate once in our experiment.

### Data for sets of Pauli gates

To verify the successful implementation of this protocol we tested its performance on a number of representative unitary gates. The first set of gates we tested were the four Pauli gates (including identity), 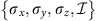. The Pauli gates have simple commutation and anti-commutation relationships: each gate only commutes with itself and identity, and anti-commutes with the other gates. For example, *σ*_*x*_ commutes with 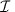 and *σ*_*x*_, and anti-commutes with *σ*_*y*_ and *σ*_*z*_. Thus, setting *U*_1_=*σ*_*x*_ means that when *U*_2_ is set to either 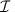 or *σ*_*x*_ the photon will always exit port 0. On the other hand, if *U*_2_ is set to *σ*_*y*_ or *σ*_*z*_, the photon should always exit port 1.

**Figure 3:**
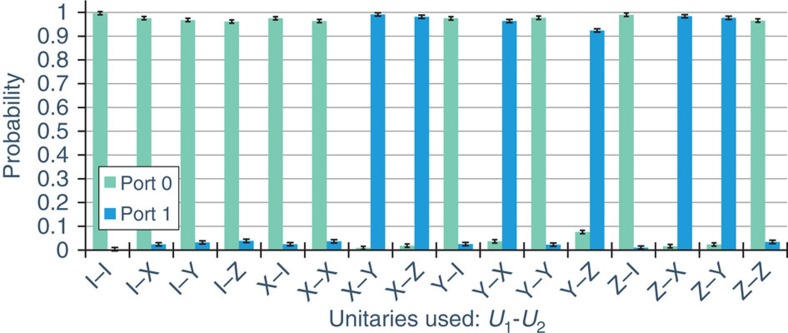
Results for Pauli Gates. Experimental data showing the probability with which the photon exits from a port when determining if a pair of random gates commute or anti-commute. The blue bars are the experimentally observed probabilities for the photon to exit port 1, and the green bars to exit port 0. If the gates commute, then, ideally, the photon should always exit port 0, while if they anti-commute the photon should exit port 1. The *x* axis is labelled with the choice of *U*_1_ and *U*_2_, where 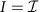, is the identity, X=*σ*_*x*_, Y=*σ*_*y*_, and Z=*σ*_*z*_. The average success rate (probability to exit the ‘correct port') of these data is 0.973±0.016.

To acquire data, *U*_1_ and *U*_2_ were first set to identity so that the phase of the interferometer could be set to *π*; this was carried out using a piezo-driven mirror (in Fig. 2). Then *U*_1_ and *U*_2_ could be set to any desired single-qubit unitary gate by setting the waveplate angles appropriately (See Supplementary Note 1 and Supplementary Table 1). For the Pauli-gate data, we cycled *U*_1_ and *U*_2_ through all 16 possible permutations and monitored the photon counts out of each port of the final beamsplitter. For every Pauli-gate combination, the probability for the photons to exit each port was estimated (see the Methods Section for details, including a discussion of the error bars). The resulting probabilities are plotted in Fig. 3. When the gates commute, we expect all of the photons to exit port 0, while if they anti-commute they should all exit port 1. Our observed data agrees very well with this prediction. For the Pauli gates, we were able to successfully determine whether a pair of gates commuted or anti-commuted with a success rate (probability to exit the ‘correct port') of 0.973±0.016. For this data, the initial polarization state was 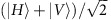, but, as we verified experimentally, the protocol is independent of the polarization (see Supplementary Note 2 and Supplementary Fig. 1).

### Comparison with the best fixed-order quantum circuit

**Figure 4:**
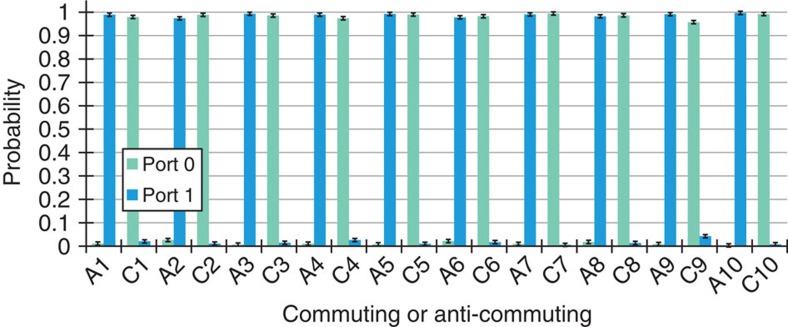
Results for Random Gates. Experimental data showing the probability with which the photon exits from a port when determining if a pair of random gates commute or anti-commute. 50 commuting and 50 anti-commuting pairs of gates were tested, of which 10 for each case are shown here. The full data set is presented in the Supplementary Fig. 2. The data representation in this figure follows the same convention as in Fig. 3. However, here the *x* axis is labelled A*i* for anti-commuting case number *i*, and C*i* for commuting case number *i*.

Without using a superposition of different quantum circuits, it is impossible to perfectly determine if two gates commute or anti-commute with a single use of each gate^8^. However, it has not been appreciated before that, using a fixed-order quantum circuit, this task can be accomplished probabilistically. In the Supplementary Note 3 we present a calculation, based on the ‘quantum comb' formalism^33^,^34^,^35^, showing that the maximum average success rate is 0.9288 (the full derivation is explicitly made another publication^36^). To rigorously compare our protocol to such a quantum circuit, we randomly generated 50 pairs of commuting gates and 50 pairs of anti-commuting gates (see the Methods Section D) and we tested our protocol with each pair. A subset of the data for these gates is plotted in Fig. 4, and the full data set in presented in Supplementary Fig. 2. The success rate of our protocol over the 100 pairs of commuting and anti-commuting gates is 0.976±0.015, which surpasses the fixed-order bound by more than three s.d.

## Discussion

Our experiment also has implications, in the broader context, for the study of causal structures in quantum mechanics, a topic that has recently received increasing theoretical^6^,^7^,^37^ and experimental^38^,^39^ attention. Traditionally, space-time events are defined with respect to some coordinate system, which describes a given underlying space-time. If we attach some fixed space-coordinates to the waveplates of our interferometer, one can say that there are two different times at which each photon can undergo the corresponding operation. In this perspective, one would describe the experiment in terms of four space-time events (in our experiment these would be: *U*_1_ first, *U*_2_ second, *U*_1_ second and *U*_2_ first), whose causal order is determined by the underlying classical space-time. Note, however, that any attempt to physically distinguish the two times at which a photon can pass through a gate would reveal which-way information and thus destroy the interference. The results of the experiment confirm that such information is not available anywhere and that the interpretation of the experiment in terms of four, causally-ordered events cannot be given any operational meaning. If, on the other hand, one requires events to be defined operationally, in terms of measurable interactions with physical systems (as in the hypothetical photon-counter example discussed above), then the experiment should be described in terms of only two events—a single use of each of the two gates. Thus we can take the interference in our experiment to demonstrate that the two events cannot be ordered according to any definite causal relation; in this sense, our experiment can be seen as the first realization of a ‘superposition of causal orders'. Indeed, it is shown elsewhere^36^ that the 2-SWITCH realized here corresponds to an example of a causally non-separable process, as defined in ref. 5.

In conclusion, we have shown how to use additional degrees of freedom of the physical system encoding a quantum system to apply quantum operations without a fixed, definite causal order. This allowed us to accomplish a task which is impossible with a fixed order of operations. Our demonstration of superimposed quantum circuits illustrates that removing the requirement of a fixed-gate order can provide quantum algorithms with real practical advantages and it shows a feasible way to obtain this advantage.

## Methods

### Single-photon source

The source generated photon pairs, in a separable polarization state, by means of the process of spontaneous parametric down conversion using a Sagnac loop^40^,^41^. The Sagnac loop was built using a dual-wavelength polarizing beamsplitter and two mirrors. A type-II collinear periodically-poled Potassium Titanyl Phosphate crystal of length 20 mm was placed inside the loop and pumped by a 23.7 mW diode laser centred at 395 nm. Photon pairs were created at degenerate wavelength 790 nm. We set the pump beam polarization to be horizontal to generate the down-converted photons in a separable polarization state 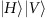. The dichroic mirror transmited the pump beam and reflected the down-converted photons, and the half waveplate (HWP) and quarter waveplate (QWP) were used to adjust the polarization of the pump beam. Long and narrow band pass filters blocked the pump beam and selected the desired down-converted wavelength. Polarizers were aligned to transmit only down-converted photons with the desired polarization. After this, the down-converted photon pairs were coupled into single-mode fibres, and one photon from the pair was used as a herald while the other single photon was sent to our interferometer using a fibre collimator.

### Optical implementation

We implemented our protocol using a Mach–Zehnder interferometer with two symmetric arm loops, see Fig. 2b. After the first beamsplitter, the reflected and transmitted beams were sent to a different combination of waveplates which formed the unitary gates. The symmetric loops were built to have the same input direction through the unitary gates for both the transmitted and reflected beams; that is, the photons always traversed the waveplates in the same direction, regardless of whether they saw *U*_1_ or *U*_2_ first. After this, the two paths were recombined on the second beam splitter. For the reflected beam, a HWP at 0° was used before the waveplates implementing *U*_2_ to ensure that the polarization state of the reflected and transmitted paths was the same before the unitary gates; this was required because the reflected path picked up a reflection phase not seen by the transmitted mode. Another a HWP at 0° was used in the transmitted arm after *U*_2_ to compensate the reflection from the second beamsplitter.

After the interferometer, the photons exiting port 0 and 1 were coupled into single-mode fibres. Then, the single photons were detected using avalanche photodiodes which were connected to a home-built coincidence counter based on Spartan 3E FPGA to register two-photon events between either port and the heralding photon. Since the coupling efficiency of each port and the detection efficiency of each avalanche photodiodes were slightly different we had to correct for this to calculate the probabilities reported in Figs 3 and 4. To perform this correction, we varied the phase of the interferometer to send all of the photons from port 0 to port 1, and we recorded the counts out of port 0 (*C*_0_) and the counts out of port 1 (*C*_1_) as the phase was varied. We then computed an efficiency factor *η*, such that *C*_0_+*C*_1_/*η* was constant. We found that *η* was typically around 0.7, but its exact valued varied because the coupling efficiencies changed slightly from one day to the next. Using this, we estimated the probability to exit port 0 as *P*_0_=*C*_0_/(*C*_0_+*C*_1_/*η*), and the probability to exit port 1 as *P*_1_=1−*P*_0_.

The visibility of the interferometer was 99.4±0.2 with a phase drift <9 mrad min^−1^. A complete data set (20 waveplate settings) was acquired in ∼2 min (1 s of data was taken at each setting, so the majority of the time was spent moving waveplates). The observed phase drift would lead to a negligible error (of only ≈0.02%) over the 2-min measurement time. At each setting ∼40,000 photon pairs were observed. The error bars were estimated by performing each measurement five times and observing the s.d.. Each measurement setting had a slightly different s.d., but for convenience we took the largest s.d. as the error bar for each measurement setting. The dominant contribution to these fluctuations was a phase drift caused by rotating the waveplates, and the Poissonian error bars (due to 40,000 counts) are much smaller than these observed fluctuations.

### Polarization unitary gates

It is well known that the combination of three waveplates (in a quarter-half-quarter configuration) can implement an arbitrary single-qubit polarization gate. Since this method is completely general, we used it to implement each of the two unitary gates *U*_1_ and *U*_2_. Each of the six waveplates were mounted using a motorized rotation mount, which allowed the unitary gates to be set remotely while only minimally disturbing the phase of the interferometer. However, we still found a slight systematic phase drift when the waveplates were rotated. We attributed this to slightly ‘wedged' shaped waveplates, which could change the optical path of each interferometer arm differently. For our waveplates (true-zero order waveplates from Special Optics, which were the most parallel waveplates we tested), we observed a maximum phase drift of 0.002 rad per deg of waveplate rotation. The waveplate angles used to implement the different polarization gates are tabulated in Supplementary Tables 1 and 2. On average, setting the six waveplates to implement a specific *U*_1_ and *U*_2_ required a total rotation of ≈45°; this would introduce a systematic error in the phase of the interferometer of ∼0.009 rad and an additional error onto our measured probabilities of 0.4%.

### Generating commuting and anti-commuting pairs of gates

We tested our protocol on 50 randomly chosen pairs of commuting gates and on 50 randomly chosen pairs of anti-commuting gates. To generate these gates we followed a very simple protocol. First, we randomly chose a unitary gate 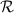 from the Haar measure. Next, to generate two anti-commuting gates *A*_1_ and *A*_2_, we set *A*_1_=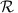
*σ*_*z*_
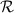
^†^ and *A*_2_=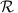
*σ*_*y*_
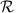
^†^ (where *σ*_*y*_ and *σ*_*z*_ are Pauli gates). Then, two commuting gates were defined from 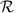 as


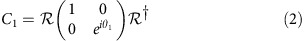


and


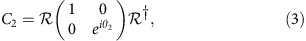


where *θ*_1_ and *θ*_2_ were also chosen randomly (we selected them from a uniform distribution between 0 and 2*π*). After this we computed the waveplate angles required to implement *C*_1_, *C*_2_, *A*_1_ and *A*_2_ (these angles are listed Supplementary Table 2).

## Additional information

**How to cite this article:** Procopio, L. M. *et al*. Experimental superposition of orders of quantum gates. *Nat. Commun.* 6:7913 doi: 10.1038/ncomms8913 (2015).

## Supplementary Material

Supplementary Information Supplementary Figure 1-3, Supplementary Tables 1-2, Supplementary Notes 1-3 and Supplementary References
