# Supplementary material for: Experimental superposition of orders of quantum gates
Source: Nat Commun. 2015 Aug 7;6:7913. doi: 10.1038/ncomms8913 (PMC4918346; doi:10.1038/ncomms8913)
Supplement: Supplementary Information — Supplementary Figure 1-3, Supplementary Tables 1-2, Supplementary Notes 1-3 and Supplementary References [file ncomms8913-s1.pdf]

SUPPLEMENTARY FIGURES

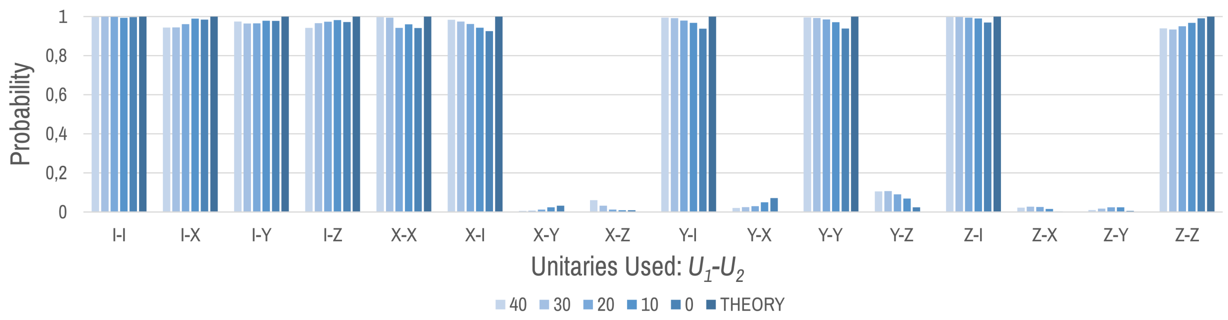

Supplementary Figure 1: State Dependent Results – Experimental data for determining if two Pauli gates commute or anti-commute for several different input states. As in Fig. 3 of the main text, the x-axis is labelled with the choice of U_1 and U_2 (where I= $\mathcal{I}$ is the identity, X= σ_x, Y= σ_y, and Z= σ_z) and the y-axis is the probability for the photon to exit port 0. If the photon exits port 0, U_1 and U_2 commute, and if it exits port 1 they anti-commute. For clarity only the port 0 probabilities are shown here. The bars of different shades are the data for different input states; the legend lists the angle of the state-preparation half waveplate, which was used to set the input state. The average success rate of these data is 0.970 ± 0.024.

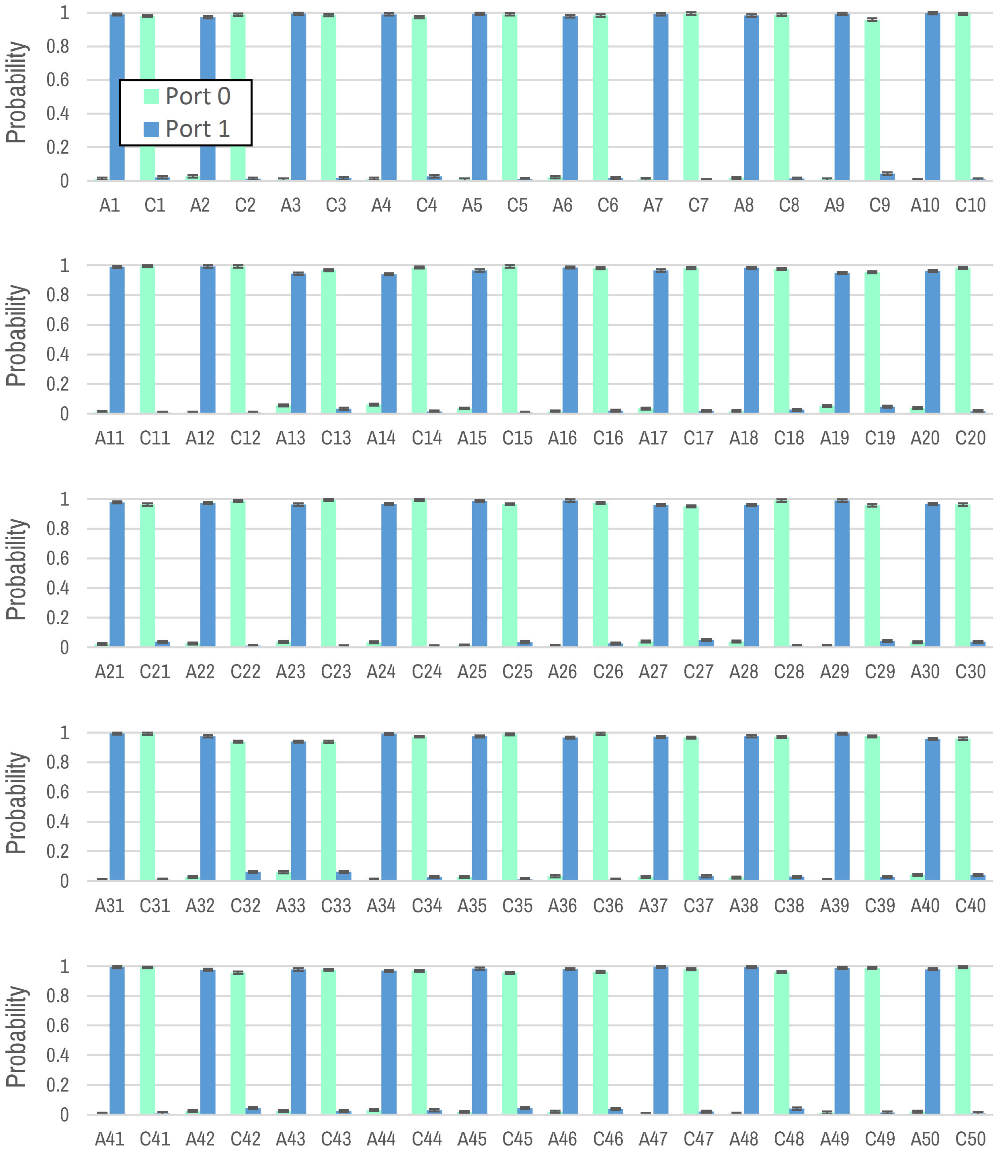

Supplementary Figure 2: Complete Set of Results for Randomly Chosen Gates – Experimental data for determining if two random gates commute or anti-commute for 100 different pairs of unitary gates. As in Fig. 4 of the main text, the x-axis is labelled with Ai for anti-commuting case number i, and Ci for commuting case number i. For clarity, only one port is shown here. The average success rate of these data is 0.976 ± 0.015.

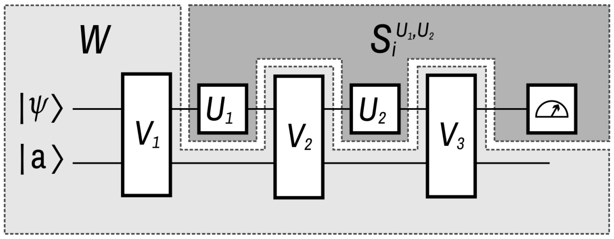

Supplementary Figure 3: Illustration of the Optimal Fixed-Order Circuit – Circuit representation of the operators W and $S_i^{U_1,U_2}$. Here $|a\rangle$ is an ancilla of arbitrary dimension, and V_1,V_2, and V_3 are arbitrary unitary gates acting on the system and the ancilla.

SUPPLEMENTARY TABLES

	Angles for U_1			Angles for U_2		
U	Q1	H1	Q2	Q3	H2	Q4
$\mathcal{I}$	0.0	0.0	0.0	0.0	0.0	0.0
σ_x	0.0	45.0	0.0	0.0	45.0	0.0
σ_y	90.0	45.0	0.0	45.0	0.0	-45.0
σ_z	90.0	0.0	0.0	0.0	0.0	90.0

Supplementary Table 1: Pauli Gate Waveplate Angles – The waveplate angles used to implement the four Pauli gates. The first column lists the desired unitary gate, the next three columns list the waveplate angles (quarter waveplate, half waveplate, then quarter waveplate) that were used to implement the gate for U_1, and the final three columns list the waveplate angles that were used for U_2.

	Commuting Angles						Anti-Commuting Angles					
	C_1			C_2			A_1			A_2		
Index	Q1	H1	Q2	Q3	H2	Q4	Q1	H1	Q2	Q3	H2	Q4
1	25.61	5.20	24.38	24.97	25.80	25.02	69.99	70.74	-20.01	110.81	70.89	20.81
2	25.64	23.90	53.26	47.93	49.85	30.96	84.45	65.67	-5.55	61.93	83.23	-28.07
3	79.12	47.13	39.88	107.12	41.53	11.89	104.50	41.47	14.50	89.83	73.62	-0.17
4	2.27	8.62	6.33	-17.71	32.37	26.31	49.30	61.80	-40.70	100.93	114.02	10.93
5	106.91	26.73	-15.24	42.54	48.87	49.12	90.83	24.37	0.83	62.14	48.83	-27.86
6	138.45	73.49	-30.11	49.73	38.22	58.61	99.17	182.20	9.17	88.31	35.83	-1.69
7	30.10	34.04	23.82	40.93	50.55	12.99	71.96	60.23	-18.04	91.00	121.56	1.00
8	69.12	32.05	25.94	21.59	64.82	73.47	92.53	26.91	2.53	88.40	67.93	-1.60
9	17.17	29.40	4.78	10.68	9.93	11.28	55.98	47.99	-34.02	91.23	115.91	1.23
10	66.71	29.40	20.31	107.06	28.21	-20.04	88.51	25.23	-1.49	53.48	46.39	-36.52
11	118.60	105.98	-34.42	35.79	148.00	48.39	87.09	6.78	-2.91	107.73	75.27	17.73
12	13.17	14.76	30.13	78.31	40.49	-35.02	66.65	41.68	-23.35	67.14	87.17	-22.86
13	29.19	51.62	15.89	19.52	4.35	25.56	67.54	63.47	-22.46	108.32	41.21	18.32
14	39.98	34.80	36.36	39.66	35.38	36.67	83.17	7.23	-6.83	49.31	31.63	-40.69
15	31.87	44.00	41.97	31.16	44.93	42.68	81.92	9.33	-8.08	74.76	47.59	-15.24
16	-7.47	30.83	25.75	-24.11	38.00	42.39	54.14	69.19	-35.86	75.63	49.82	-14.37
17	47.80	81.82	-2.10	22.27	24.28	23.43	67.85	78.89	-22.15	65.59	31.66	-24.41
18	17.57	24.35	12.72	54.18	52.86	-23.89	60.15	53.01	-29.85	97.45	30.56	7.45
19	18.30	145.88	-53.14	1.14	61.19	-35.98	117.58	90.00	27.58	78.13	81.95	-11.87
20	24.36	29.96	10.69	54.25	48.53	-19.20	62.53	49.02	-27.47	119.72	100.21	29.72
21	81.68	4.23	38.15	148.16	66.28	-28.34	104.91	187.37	14.91	48.04	19.93	-41.96
22	-12.21	-6.83	-9.66	-43.49	24.68	21.61	124.06	115.50	34.06	97.12	38.32	7.12
23	23.49	27.69	-38.97	167.49	68.11	-2.96	37.26	28.70	-52.74	68.18	6.62	-21.82
24	51.36	52.70	49.32	46.18	41.08	54.50	95.34	83.67	5.34	82.13	24.26	-7.87
25	-11.32	79.67	25.49	51.83	31.71	-37.66	52.08	31.71	-37.92	83.82	7.52	-6.18
26	-7.62	41.69	28.74	0.43	34.57	20.70	55.56	64.22	-34.44	122.61	65.55	32.61
27	28.05	6.10	11.74	52.45	80.22	-12.66	64.89	79.02	-25.11	35.26	103.48	-54.74
28	77.86	19.97	23.19	72.41	22.05	28.64	95.52	17.64	5.52	83.99	52.03	-6.01
29	58.96	81.60	-17.09	23.84	16.07	18.03	65.93	81.22	-24.07	60.13	30.68	-29.87
30	18.74	38.29	19.72	19.58	4.53	18.88	64.23	64.85	-25.77	109.50	31.32	19.50
31	48.00	37.76	-13.85	21.01	20.97	13.14	62.08	39.56	-27.92	59.53	81.95	-30.47
32	7.79	60.78	-18.10	0.78	70.97	-11.09	129.84	119.16	39.84	82.52	76.68	-7.48
33	100.79	84.36	-37.71	41.77	0.53	21.32	76.54	81.80	-13.46	29.01	41.72	-60.99
34	-2.47	-18.24	-20.91	-43.90	5.08	20.52	123.31	96.47	33.31	50.29	34.60	-39.71
35	70.25	59.09	35.26	-40.49	54.02	-34.00	97.75	63.46	7.75	108.68	118.68	18.68
36	82.08	94.57	48.90	126.39	99.59	4.59	110.49	101.10	20.49	95.66	39.98	5.66
37	59.51	57.89	41.49	33.06	37.51	67.94	95.50	-19.28	5.50	50.65	50.77	-39.35
38	7.54	6.90	8.93	-17.77	69.99	34.24	53.23	39.46	-36.77	95.20	10.93	5.20
39	8.81	57.10	37.25	18.95	41.33	27.11	68.03	73.44	-21.97	103.03	72.63	13.03
40	20.74	40.54	17.97	18.33	2.30	20.39	64.36	62.84	-25.64	109.31	20.23	19.31
41	58.10	32.61	40.43	50.68	46.22	47.85	94.27	106.66	4.27	35.45	-31.98	-54.55
42	31.72	1.79	29.83	30.28	50.47	31.26	75.77	76.36	-14.23	30.02	55.96	-59.98
43	22.65	31.45	28.14	40.86	0.87	9.93	70.40	82.42	-19.60	62.93	30.32	-27.07
44	37.95	125.41	51.23	57.55	60.72	31.62	89.59	72.23	-0.41	63.13	83.65	-26.87
45	39.99	58.07	14.13	26.84	26.10	27.28	72.06	65.53	-17.94	84.90	32.69	-5.10
46	13.21	24.69	1.16	5.26	0.84	9.10	52.18	43.89	-37.82	88.55	20.99	-1.45
47	80.68	89.06	-29.71	1.99	48.40	48.97	70.48	88.17	-19.52	100.80	83.49	10.80
48	94.56	59.52	-31.27	39.31	44.44	23.99	76.65	62.20	-13.35	129.97	116.26	39.97
49	30.87	36.27	60.40	52.65	50.37	38.62	90.64	62.90	0.64	-51.99	43.14	38.01
50	39.84	42.78	41.17	49.05	17.95	31.97	85.51	3.66	-4.49	85.87	49.02	-4.13

Supplementary Table 2: Random Gate Waveplate Angles – The waveplate angles used to implement the 100 randomly chosen commuting and anti-commuting gates.

Supplementary Note 1. WAVEPLATE SETTINGS

Each unitary gate that we tested was implemented with three waveplates: first a quarter waveplate, then a half waveplate, followed by a final quarter waveplate. Thus we need a total of six waveplates for two unitary gates. Each waveplate was also mounted in a motorized rotation mount, allowing it to be remotely set without disturbing the phase of the interferometer.

For completeness, here we list the waveplate settings used to implement the various unitary gates that we tested. Supplementary Table 1 lists the waveplate angles for the four Pauli gates. For experimental convenience, we used different angles to set U_1 and U_2 to σ_y and σ_z. This has no effect on the logical gate that the waveplates apply. In fact, this can be seen as a further verification of our protocol: the protocol's success is independent of the physical apparatus used to enact each gate.

The next set of 100 gates that we tested were randomly generated commuting and anti-commuting gates. As described in the Methods Section, we first randomly generate a gate $\mathcal{R}$. Two anti-commuting gates A_1 and A_2 were generated from $\mathcal{R}$ by setting $A_1 = \mathcal{R}\sigma_z\mathcal{R}^\dagger$ and $A_2 = \mathcal{R}\sigma_y\mathcal{R}^\dagger$ (where σ_y and σ_z are Pauli gates). Two commuting gates were constructed as

$$C_1 = \mathcal{R}\begin{pmatrix} 1 & 0 \\ 0 & e^{i\theta_1} \end{pmatrix}\mathcal{R}^\dagger \tag{1}$$

and

$$C_2 = \mathcal{R}\begin{pmatrix} 1 & 0 \\ 0 & e^{i\theta_2} \end{pmatrix}\mathcal{R}^\dagger \tag{2}$$

where θ_1 and θ_2 are chosen randomly between 0 and 2π from a uniform distribution. The waveplate angles that we used to implement these 100 pairs of gates are tabulated in Supplementary Table 2.

Supplementary Note 2. ADDITIONAL RAW DATA

As we discussed in the main text, our protocol to determine if U_1 and U_2 commute or anti-commute is independent of the state of qubit 2 (defined in Eq. 1 of the main text).

In our experiment, qubit 2 is encoded in the polarization state of our single photons. As an additional verification of our implementation we also tested the state dependence of our protocol experimentally. To do this, we simply changed the state of qubit 2, by rotating the state-preparation half waveplate, and repeated our protocol for several different input states. We rotated this HWP in steps of 10°, from 0° to 40° and took "Pauli-gate data" (described in the main text) for each waveplate setting. These data are plotted in Supplementary Figure 1. There were no significant changes to the performance of our protocol for any of these settings, and the success rate averaged over these five states was 0.970 ± 0.024. In addition to the data presented in the main text, we verified our protocol's operation on 50 different pairs of commuting gates and 50 different pairs of anti-commuting gates. The 50 different cases were taken in groups of 10, and the data for each group was acquired in the same way as the Pauli gates. Before acquiring each group data, the phase of the interferometer was set to π while U_1 and U_2 were set to implement identity. Then U_1 and U_2 were set to implement some other gates, and photons were counted for 1 second on each setting. These data are presented in Supplementary Figure 2, and they exhibit an average success rate of 0.976 ± 0.015.

Supplementary Note 3. A FIXED-ORDER QUANTUM CIRCUIT FOR OUR TASK

In [1] it was proven that quantum circuits with a fixed gate order (fixed-order circuits) cannot distinguish between commuting and anti-commuting unitary gates with probability one under the condition that each unitary is queried once. Theoretically, exploiting a superposition of different gate orders allows for the two cases to be distinguished perfectly under the same condition. However, since an experimental implementation of the superposition necessary involved errors, one should compare our experiment to a fixed-order circuit which can probabilistically distinguish between the two cases. Here we will show that our experimental success probability is higher than the maximal probability of success of any fixed-order circuit.

We will assume that to determine if two gates commute or anti-commute we will run a fixed-order circuit that contains a single copy of U_1 and U_2 and afterwards measure a single qubit. If the result of this single-qubit measurement is zero (one) we will say the two gates commute (anticommute). Then, the probability of success is defined as the average of the

probability of measuring zero given that the unitary gates anti-commute and the probability of measuring one given that the unitary gates commute. In symbols:

$$p_{\text{succ}} = \frac{1}{2}(p(0|C) + p(1|A)). \tag{3}$$

To calculate p_{succ}, we use tools from [2–4], and the full calculation is presented in Ref. [5], Section V. One can separate any circuit that is applied to determine if the unitaries commute or anticommute in two parts: the first is represented by an operator

$$S_i^{U_1,U_2} = \mathfrak{C}(U_1) \otimes \mathfrak{C}(U_2) \otimes |i\rangle\langle i|, \quad i = 0, 1 \tag{4}$$

that represent the unitaries U_1 and U_2 and the outcome i of a measurement in the Z basis, where $\mathfrak{C}(U_j)$, with $j = 1, 2$, is the Choi-Jamiołkowski operator of the unitary U_j. The second is represented by an operator W that represents the most general fixed-order circuit (or even a convex combination of fixed-order circuits) that connects the unitaries and the measurement. A circuit representation of these operators is shown in Supplementary Figure 3. In this case,

$$p(i|U_1, U_2) = \operatorname{tr}(S_i^{U_1,U_2} W) \tag{5}$$

is the probability of obtaining outcome i after applying unitaries U_1 and U_2.

The probabilities $p(0|C)$ and $p(1|A)$ are then the averages

$$\begin{aligned} p(0|C) &= \int \mathrm{d}\mu_C p(0|U_1, U_2) \\ &= \operatorname{tr}\left[\left(\int \mathrm{d}\mu_C S_0^{U_1,U_2}\right) W\right]. \end{aligned} \tag{6}$$

$$\begin{aligned} p(1|A) &= \int \mathrm{d}\mu_A p(1|U_1, U_2) \\ &= \operatorname{tr}\left[\left(\int \mathrm{d}\mu_A S_1^{U_1,U_2}\right) W\right]. \end{aligned} \tag{7}$$

where $\mathrm{d}\mu_C, \mathrm{d}\mu_A$ are measures on the set of commuting and anticommuting pairs of unitaries, respectively. One should note, now, that p_{succ} depends crucially on the choice of measures $\mathrm{d}\mu_C, \mathrm{d}\mu_A$. For instance, if one selects U_1 and U_2 only from the Pauli matrices, then $p_{\text{succ}} = 1$.

We chose measures, shown in the Methods section, that can generate any pair of commuting or anticommuting unitaries, modulo a global phase. Defining the operators

$$S_0^C = \int \mathrm{d}\mu_C S_0^{U_1,U_2} \tag{8}$$

$$S_1^A = \int \mathrm{d}\mu_A S_1^{U_1,U_2}, \tag{9}$$

we have finally that the probability of success can be expressed as

$$p_{\text{succ}} = \mathrm{tr}[W \left(S_0^C + S_1^A\right)/2], \tag{10}$$

which is a linear function of W. Since one can characterize the set of fixed-order circuits through linear constraints on positive semidefinite operators, optimizing p_{succ} is a semidefinite program (SDP), which can be guaranteed to reach the global optimum [2–5]. Solving this optimization problem numerically, we found that

$$p_{\text{succ}} = 0.9288, \tag{11}$$

which is significantly lower than the success probability of 0.976 ± 0.015 that we measured experimentally.

As an extra verification, we tested the specific 100 pairs of gates that we used in our experiment with the optimal fixed-order circuit. We found that this optimal fixed-order circuit has success rate of 0.9390 averaged on these 100 pairs of gates – still much lower than our experimental success probability.

SUPPLEMENTARY REFERENCES

[1] Giulio Chiribella, "Perfect discrimination of no-signalling channels via quantum superposition of causal structures," Physical Review A **86**, 040301 (2012).

[2] G. Chiribella, G. M. D'Ariano, and P. Perinotti, "Quantum Circuit Architecture," Physical Review Letters **101**, 060401 (2008).

[3] Gus Gutoski and John Watrous, "Toward a general theory of quantum games," in *Proceedings of the thirty-ninth annual ACM symposium on Theory of computing* (ACM, 2007) pp. 565–574.

[4] Giulio Chiribella, "Optimal networks for quantum metrology: semidefinite programs and product rules," New Journal of Physics **14**, 125008 (2012).

[5] Mateus Araújo, Cyril Branciard, Fabio Costa, Adrien Feix, Christina Giarmatzi, and Časlav Brukner, "Witnessing causal nonseparability," Preprint at http://arxiv.org/abs/1506.03776 (2015).
